# Urinary cytology: a potential tool for differential diagnosis of acute kidney injury in patients with nephrotic syndrome

**DOI:** 10.1186/s13104-020-05244-6

**Published:** 2020-08-27

**Authors:** Caroline Vilas Boas de Melo, Maria Brandão Tavares, Paula Neves Fernandes, Carlos Alberto dos Santos Silva, Ricardo David Couto, Marília Bahiense Oliveira, Washington L. C. dos-Santos

**Affiliations:** 1 Fundação Oswaldo Cruz, Instituto Gonçalo Moniz, Rua Waldemar Falcão 121, Candeal, Salvador, BA CEP 40296-710 Brazil; 2 Escola Bahiana de Medicina e Saúde Pública, Salvador, BA Brazil; 3 Medicine Department, Federal University of Bahia, Salvador, BA Brazil; 4 Pharmaceutic Department, Federal University of Bahia, Salvador, BA Brazil; 5 Nephrology Department, Ana Nery Hospital, Salvador, BA Brazil

**Keywords:** Acute tubular necrosis, Glomerulonephritis, Cytodiagnosis, Acute kidney injury

## Abstract

**Objective:**

Acute tubular necrosis (ATN) is a frequent cause of acute kidney injury (AKI). In patients with nephrotic syndrome (NS), AKI demands the differential diagnosis between ATN and rapidly progressive glomerulonephritis. In some cases, conclusive diagnosis is possible only by kidney biopsy. We aimed to study the potential use of urine cytology in the differential diagnosis between ATN and proliferative glomerular lesion in patients with NS.

**Results:**

Cell size analysis showed a higher proportion of small cells and a lower proportion of large cells in the urine of patients with AKI. Cells phenotypes were easily defined using cytological preparations. Leukocytes were found to be a primary classifier of NS groups, with higher number in patients with AKI and patients with proliferative glomerular lesions. Although renal biopsy is still required for confirmative diagnosis, our data suggests that urinary cytology can be readily performed and support the differential diagnosis between proliferative glomerular lesion and ATN in patients with NS and AKI.

## Introduction

Acute tubular necrosis (ATN) is a leading cause of acute kidney injury (AKI) in hospitalized patients [1]. The prevalence of AKI also correlates with ATN severity in patients with nephrotic syndrome (NS) [2]. Emergence of AKI in patients with NS requires the differential diagnosis between ATN alone and glomerular proliferative lesions such as in crescentic glomerulonephritis [3], since the therapeutic approach differs between these conditions. Whereas proliferative glomerulopathies require immediate immunosuppression to avoid progression to end-stage renal disease, ATN requires support treatment without immunosuppression avoiding potential side effects [4]. Urinary sediment analysis has been used in the diagnosis of ATN and in the differential diagnosis between isolated ATN and proliferative glomerular lesion in patients with AKI [5, 6]. The presence of renal tubular epithelial cells in the urinary sediment has been considered indicative of ATN, and high number of leukocytes has been considered indicative of glomerular lesions [5–7]. However, cell identification in unstained urine sediment can be difficult, particularly in conditions of glomerular lesions associated with nephrotic syndrome, where the urinary sediment may be complex. Furthermore, studies associating urinary sediment cytological findings with kidney histology are lacking. In this work, we compare the urinary cytology with the histological presentation of the kidney in biopsy of patients with NS and AKI. To avoid inconsistent cell identification, we used conventional staining techniques used in cytology. Our data suggest that conventionally stained urine cytology can be a rapid, consistent and easy-to-use tool for supporting the differential diagnosis between proliferative glomerular lesion and ATN in patients with NS and AKI. We propose a cross-validation of this model to support larger studies for validation of the test in the bedside.

## Main text

### Methods

#### Patients

A prospective cross-sectional study including 27 patients with NS subjected to renal biopsy for glomerular disease diagnosis in referral hospitals of Salvador, Brazil, from July 2013 to April 2015. The biopsies were examined at the Fundação Oswaldo Cruz, Instituto Gonçalo Moniz in Salvador, Brazil. Cases were excluded if the renal biopsy contained less than 7 glomeruli, if the estimated interstitial fibrosis encompassed 30% or more of the cortical area, if the patient had diabetes mellitus or infections. The patients were allocated into 3 groups: PRO—8 patients with proliferative glomerulopathy, ATN—10 patients with ATN without proliferative glomerulonephritis and Non-ATN—9 patients without ATN or proliferative glomerular lesions. AKI was defined using the KDIGO criteria. Ten healthy volunteers were used as a reference group of normal urine cytology.

#### Clinical data

The following data were obtained from biopsy request forms and by anamneses: age, sex, serum creatinine, albumin, cholesterol, 24-h urine proteinuria and diagnosis of systemic arterial hypertension.

#### Histological analysis

The renal specimens were obtained by percutaneous biopsies, fixed in Bouin’s solution or acid formalin, paraffin embedded, cut into 2-μm thick sections, and stained with hematoxylin and eosin. All the slides were reviewed by a pathologist (WLCS). The intensity of ATN was estimated as a percentage of the renal cortex by visual assessment. The following tubular changes were considered as evidence of either current or recent ATN: tubular dilatation, thinning of the tubular epithelium, cellular casts, interstitial edema, and the evidence of epithelial regeneration (hyperchromatic nucleus, mitosis, and binucleation). The percentage of cortical tubulointerstitial fibrosis was estimated by visual assessment.

#### Cytology analysis

Fresh urine was obtained from the patients by spontaneous voiding before renal biopsy. Ten milliliters of urine were centrifuged at 2000*g* per 10 min in a standard centrifuge. The supernatant was removed by suction. The sediment was resuspended in 100 µl of Hank’s balanced salt solution (HBSS) and cytocentrifuged onto histological slides at 500 rpm per 5 min, fixed with a methanol-based buffered preservative solution and stained with hematoxylin and eosin. Ten low-power (×100), non-overlapping images were collected from each patient’s cytological smears using a camera attached to a light microscope (CX41, Olympus, Tokyo, Japan) and Image-Pro Plus software version 7.0 (MediaCybernetics, Inc., Bethesda, MD, USA). Cells were classified as small, medium or large. Morphometric estimates of cell diameter revealed that the cells classified as small measured up to 30 µm, those classified as medium measured 30–48 µm, and those classified as large measured over 48 µm. The cells were further classified as squamous cells (large cells with irregular cytoplasm and round and central nuclei), urothelial cells (large cells with regular rounded cytoplasm), renal tubular epithelial cells (small cells with small rounded nuclei and basophilic cytoplasm) or leukocytes (small cells with lobulated or oval hyperchromatic nuclei and basophilic cytoplasm).

#### Cell immunophenotyping

Cytological preparations were fixed in cold acetone and labeled for tubular cells and leukocytes identification. KIM-1/TIM-1 antibody (Abcam 47635) was used at 5 µg/ml diluted in phosphate buffered saline (PBS) containing 1% bovine serum albumin (BSA), 10% normal goat serum, 0.3 M glycine and 0.1% Tween 20, followed by a secondary antibody conjugated to Alexa Fluor 490 (green) at 1:200. For leukocyte identification, the slides were incubated with CD45 (Abcam 27287) FITC (green) at 1:100 in 1% PBS-BSA. DAPI was used to stain the cell nuclei.

#### Prediction of model and cross-validation

Models of prediction and cross-validation were built using Orange Data Mining software (University of Ljubljana). The classification based on cell morphology and the diagnosis of AKI were tested for prediction of the patient groups. The accuracy of the model, represented by area under the ROC curve (AUC) was assessed using cross-validation between three learning models: logistic regression, random forest and tree.

#### Statistical analysis

Continuous variables are summarized as the means ± standard deviations or median and first and third quartiles and were compared using the Kruskal–Wallis or ANOVA followed by Bonferroni’s multiple comparison tests when required. Comparisons of proportions were performed using chi-square test or Fisher’s exact probability test. Principal component analysis (PCA) graph was used to illustrate multivariate analysis of the data on cell morphology. The results were considered statistically significant at P < 0.05. Data were analyzed using Prism 5.01 (GraphPad, San Diego, CA, USA) and Stata/IC 11 data analysis and statistical software (StataCorp LLC, College Station, TX, USA).

#### Ethical statement

All patients were informed about the research and agreed to participate. This work was approved by Research Ethical Committee of Fundação Oswaldo Cruz, Instituto Gonçalo Moniz, Salvador, BA, Brazil, Protocol No. 184.419.

## Results

### General patient characteristics

The main characteristics of the enrolled patients are shown in Table 1. Hypoalbuminemia was more severe in the ATN (1.7 [1.6–1.8] g/dl) than in the PRO (2.3 [1.7–2.8] g/dl) group (P = 0.04). Serum cholesterol level was significantly higher in the ATN (372 [278.5-581.8] mg/dl) than in the PRO (235 [139-291] mg/dl) group (P = 0.02) (Table 1). Although the serum concentrations of creatinine were above the reference values for the ATN (1.4 [0.7–2.0] mg/dl) and PRO (1.7 [0.7–2.0] mg/dl) groups no statistically significant differences were observed among the groups. The main diagnosis of patients of the ATN or Non-ATN group was minimal change disease (MCD) (40% and 44%, respectively), followed by focal and segmental glomerulosclerosis (FSGS) (30% and 33%, respectively). Lupus nephritis (LN) was the most frequent histological diagnosis in patients of the PRO group (50%) (Table 1). Evidence of recent ATN was found in 63% of biopsies. Seven patients of the PRO group also presented ATN.

**Table 1:** General characteristics of the patients with nephrotic syndrome and the healthy individuals enrolled in the study

Parameter	Control	Non-ATN	ATN	PRO	P value
Total (*n*)	10	9	10	8	
Gender					
Female	5 (50%)	4 (44%)	5 (50%)	5 (63%)	–
Age (years)^a^	30 [25.5–36.75]	25 [21.5–50.5]	50.5 [20–56]	25.5 [22–46.7]	ns
Serum urea (mg/dl)^a^	25 [22.75–28.9]	33 [20–47]	64 [37.5–107]	36.5 [24.2–89.5]	ns
Serum creatinine (mg/dl)^a^	0.9 [0.82–0.97]	1.1 [0.8–1.7]	1.4 [0.7–2.0]	1.7 [0.7–2.0]	ns
Serum albumin (g/dl)^a^	–	1.9 [1.6–2.2]	1.7 [1.6–1.8]	2.3 [1.7–2.8]	0.04*
Total cholesterol (mg/dl)^a^	–	360.5 [256–470]	372 [278.5–581.8]	235 [139–291]	0.02*
24-h protein (mg)^a^	–	6965 [3913–13,437]	7540 [3482–13,844]	8488 [2860–16,081]	ns
SAH	–	8 (89%)	9 (90%)	5 (63%)	ns
Histological diagnostic					
MCD	–	4 (44%)	4 (40%)	0	–
FSGS	–	3 (33%)	3 (30%)		
MN	–	2 (22%)	2 (20%)	0	–
NL	–	0	0	4 (50%)	–
DPGN	–	0	0	2 (25%)	–
MPGN	–	0	1 (10%)	2 (25%)	–
Tubulointerstitial fibrosis^b^		7.7 ± 7.9%	9.4 ± 7.6%	8.1 ± 7%	–
Acute tubular necrosis (ATN)	–	0	10 (100%)	7 (88%)	
Intensity ATN^b^	–	3.3 ± 2.5%	46 ± 26.1%	29.3 ± 26.7%	–

*SAH* systemic arterial hypertension, *MCD* minimal change disease, *FSGS* focal and segmental glomerulosclerosis, *MN* membranous nephropathy, *LN* lupus nephritis, *DPGN* diffuse-proliferative glomerulonephritis, *MPGN* membranoproliferative glomerulonephritis, *ns* not significant

* Acute tubular necrosis group (ATN) vs. inflammatory-proliferative glomerular lesion group (PRO)

^a^Data expressed as medians and interquartile intervals

^b^Data expressed as medians ± standard deviations

### Cytological analysis

The urinary sediment of one patient without ATN and of three patients with ATN did not provide enough cells for cytology analysis. Therefore, cell characteristics were studied for 23 cases.

#### Cell size

Patients with AKI showed a higher proportion of small cells (75.3 ± 18.8%) and a lower proportion of large cells (10.4 ± 11.9%) than did patients without AKI (30.7 ± 25.8%, P = 0.0007; 57 ± 27.4%, P = 0.0003, respectively) (Table 2). However, although a trend towards an increased proportion of small cells was observed in patients with proliferative glomerular lesion, this trend was not statistically significant.

**Table 2:** Estimates of cell populations in the urine of patients with nephrotic syndrome and healthy volunteers included in the study

Category	Control	NS patients		P value
		With AKI	Without AKI	
Total (*n*)	10 (%)	9 (%)	9 (%)	
Small cells	31 ± 29.9	75.3 ± 18.8	30.7 ± 25.8	0.0007*
Medium cells	4.8 ± 5	14.2 ± 9.8	12.2 ± 13	ns
Large cells	64.1 ± 30.3	10.4 ± 11.9	57 ± 27.4	0.0003*
Squamous cells	64 ± 35.1	4.2 ± 3.2	39.7 ± 23.5	0.0004*
Urothelial cells	11.3 ± 14.9	27.3 ± 21.8	33.8 ± 19.9	ns
Renal epithelial tubular cells	4 ± 5.5	26.7 ± 23.1	14.1 ± 19.6	ns
Leukocytes	21.1 ± 22.9	41.4 ± 35	12.2 ± 11.9	0.03*

Data expressed as medians of proportion ± standard deviation

*NS* nephrotic syndrome, *AKI* acute kidney injury

* Difference between groups with or without AKI

**Fig. 1:**
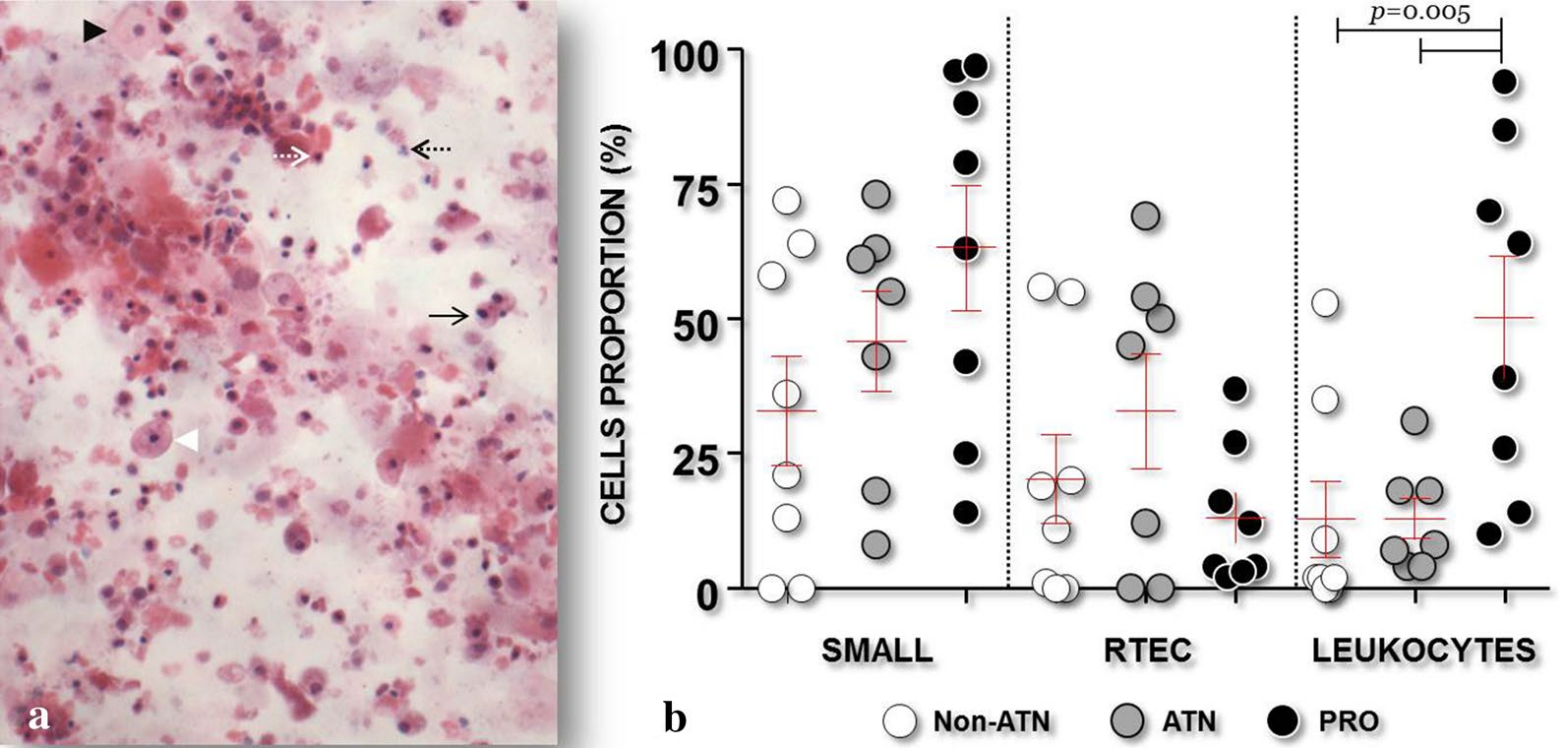
Representative photomicrograph of the urinary sediment, stained with H/E, of a patient with nephrotic syndrome (**a**) (×200). Dotted arrows identify small cells; white indicates renal tubular epithelial cell, black indicates leukocyte. Head arrows identify large cells: white indicates urothelial cell, black indicates squamous cell. Continuous arrows identify medium cells: urothelial cells. **b** Proportions of small cells, i.e., renal epithelial tubular cells (RTEC) and leukocytes, in groups without ATN (Non-ATN), with ATN (ATN) and with glomerular proliferative lesion (PRO)

Morphological identification of the cell populations: Patients with AKI showed a higher proportion of leukocytes (41.4 ± 35%) and a lower proportion of squamous cells (4.2 ± 3.2%) than did patients without AKI (12.2 ± 11.9%, P = 0.03; 39.7 ± 23.5%, P = 0.0004, respectively) (Table 2). Furthermore, patients of the PRO group showed a higher proportion of leukocytes (50.2 ± 32.4%) than did the other NS patients (Non-ATN group, 12.88 ± 20%; ATN group, 12.86 ± 9.9%; P = 0.005) (Fig. 1).

The reliability of the morphological identification was confirmed by positive immunolabeling of leukocytes with anti-CD45 antibody and of renal tubular epithelial cells with anti-KIM-1 antibody (Additional file 1: Figure S1).

#### Model of urine cytology for differential diagnosis of AKI

The tree learner model had the best performance analysis (AUC 0.864 and precision of 0.909, Additional file 2: Table S1) for prediction of AKI. Using this model for diagnosis of AKI and morphology of cells, a cluster of patients with NS was observed in the three groups as shown in the PCA analysis (Additional file 3: Figure S2A). In the binary tree model, the counts and morphology of cells were able to classify the NS groups the data set (Additional file 3: Figure S2B).

## Discussion

In this study, we analyzed the potential use of urine cytology in the differential diagnosis of AKI in patients with NS. Similar studies have been conducted with patients without NS. In NS, the high protein concentration and the presence of different proteins in the urine may interfere with cell representation in urine sediment [8]. Furthermore, urine sediment may be enriched in some patients with NS. Nevertheless, we found that small cells predominated in the urine sediment of patients with AKI. Although a trend towards an increased population of small cells was observed in patients with proliferative glomerulopathy, cell size alone could not distinguish the potential cause of AKI. This small cell population included tubular epithelial cells and leukocytes, as identified by cell morphology and confirmed morphologically and by immunofluorescence assay. Of these two cell populations, the leukocytes proportion was higher in the urine sediment of patients with proliferative glomerulopathy than in that of the remaining patient groups. Similar change has been reported in urine of patients with glomerular proliferative disease without NS [5]. The number of leukocytes was found to be a primary classifier of NS groups of patients in the learning model applied to this image dataset. Perazella et al., using phase contrast microscopy, found a significant increase in the proportion of epithelial tubular cells in patients with ATN compared with patients with pre-renal AKI [6]. Although we found a trend toward an increase in tubular epithelial cells in patients with nephrotic syndrome and ATN alone relative to the other patient groups, this difference was not statistically significant. A possible reason for the difference between studies is that patients with glomerular diseases were excluded from the study by Perazella and coworkers [6]. Patients with glomerular disease might present a more complex urinary sediment, which might affect the proportions of the different cell populations. Furthermore, histological confirmation of ATN was lacking in the study by Perazella and coworkers [6].

The staining of urinary sediment is rapid, easily performed, and inexpensive, and staining reagents are widely available. We show that this procedure allows even professionals with little experience in cytology to confirm the diagnosis of AKI and to distinguish proliferative glomerulonephritis as the potential cause of this condition in patients with the emergence of kidney dysfunction in the course of NS. The clustering of patients with the three different causes of AKI based in the number and cell morphological types and using the decision tree reported herein support further studies with a larger number of patients.

## Conclusions

1. Using urine cytology with conventional staining might constitute a helpful tool for the differential diagnosis between proliferative glomerular lesion and ATN in patients with NS and AKI in the absence of kidney biopsy.
2. The classification method based in cell number and types has potential use in the distinction of AKI etiology in patients with NS.

## Limitations

1. Although the use of urine cytology provided some direction in the differential diagnosis of AKI in patients with NS, renal biopsy is still needed for confirmation.
2. Larger sample size and different hospital settings are needed to validate urine cytology as an alternative tool for diagnosis of AKI.

## Supplementary information

**Additional file 1: Figure S1.** Representative photomicrograph of the urinary sediment stained with H/E of a patient with nephrotic syndrome (x400) (A). Immunofluorescence of the urinary sediment of an acute tubular necrosis patient showing positive marking of KIM-1 (green) and nucleus (blue) (B), and immunofluorescence of the urinary sediment of an inflammatory-proliferative glomerular lesion patient showing positive marking of CD45 (green) and nucleus (blue) (C).

**Additional file 2: Table S1.** Cross-validation of models in urine cytology for differential diagnosis of AKI.

**Additional file 3: Figure S2.** Algorithmic models of patients without ATN or proliferative glomerular disease (Non-ATN – Group 1/blue), with ATN (ATN – Group 2/red) and with glomerular proliferative lesion (PRO – Group 3/green). **(A)** Principal component analysis of groups of patients based in AKI diagnosis and cell numbers and types. **(B)** Binary tree model of groups of patients based in cell numbers and types. Transition of colors means the classification of groups accordingly as follows: blue - Non-ATN/Group 1; red – ATN/Group 2 and green – PRO/Group 3.

## Data Availability

All data obtained during this study is included in this article.

## Abbreviations

AKI: Acute kidney injury
ATN: Acute tubular necrosis
AUC: Area under the ROC curve
BSA: Bovine serum albumin
DPGN: Diffuse-proliferative glomerulonephritis
FITC: Fluorescein isothiocyanate
FSGS: Focal and segmental glomerulosclerosis
HBSS: Hank’s balanced salt solution
KIM-1/TIM-1: Kidney Injury Molecule-1/T cell immunoglobulin and mucin-containing molecule
LN: Lupus nephritis
MCD: Minimal change disease
MN: Membranous nephropaty
MPGN: Membranoproliferative glomerulonephritis
Non-ATN: Group og patients without ATN or proliferative glomerular lesions
NS: Nephrotic syndrome
PBS: Phosphate buffered saline
PCA: Principal component analysis
PRO: Group of patients with proliferative glomerulopathy
SAH: Systemic arterial hypertension
