# Supplementary figures and images for: Urinary cytology: a potential tool for differential diagnosis of acute kidney injury in patients with nephrotic syndrome
Source: BMC Res Notes. 2020 Aug 27;13:401. doi: 10.1186/s13104-020-05244-6 (PMC7453712; doi:10.1186/s13104-020-05244-6)

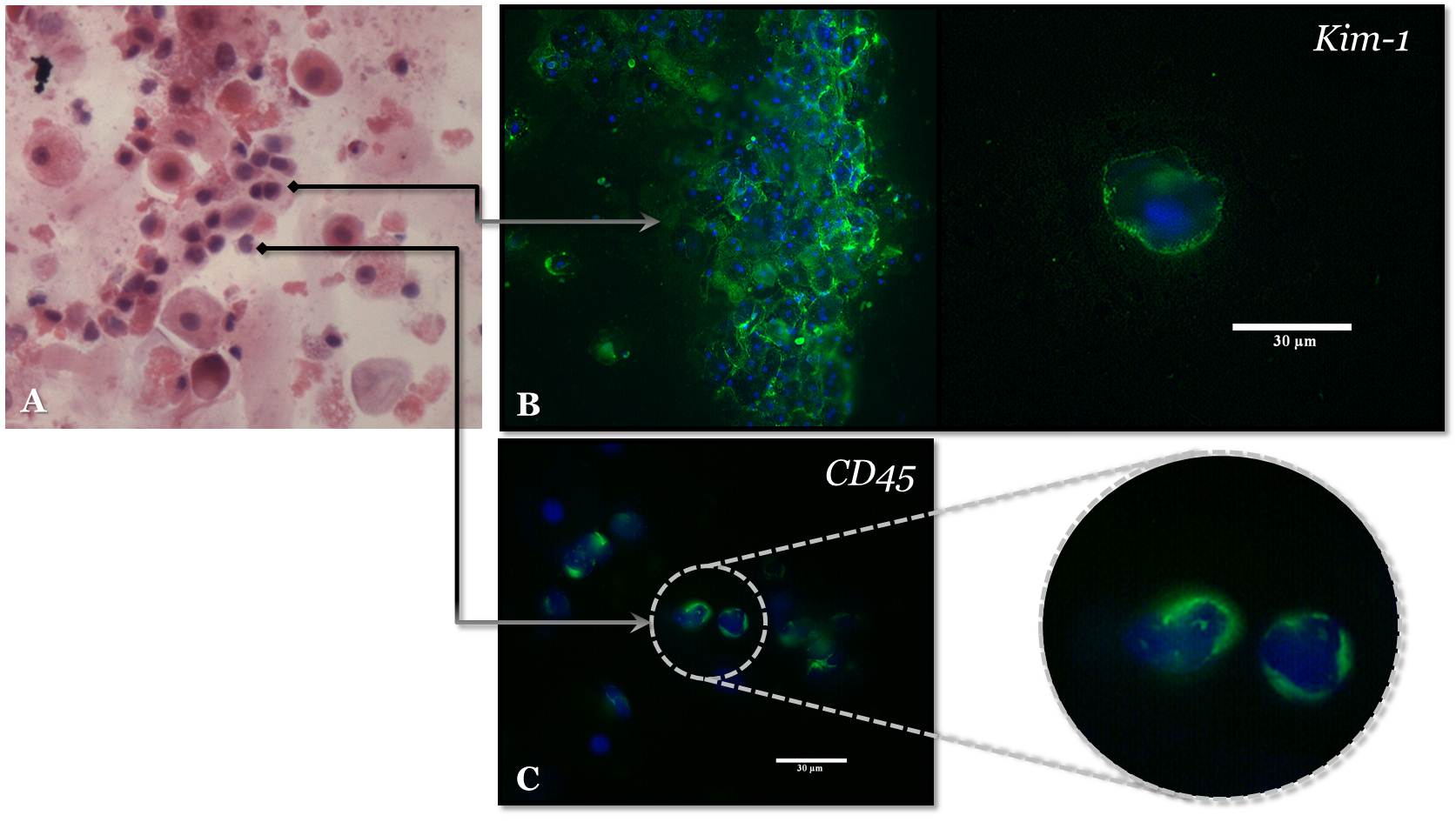

Supplement: Supplementary file 1 — Additional file 1: Figure S1. Representative photomicrograph of the urinary sediment stained with H/E of a patient with nephrotic syndrome (x400) (A). Immunofluorescence of the urinary sediment of an acute tubular necrosis patient showing positive marking of KIM-1 (green) and nucleus (blue) (B), and immunofluorescence of the urinary sediment of an inflammatory-proliferative glomerular lesion patient showing positive marking of CD45 (green) and nucleus (blue) (C). [file 13104_2020_5244_MOESM1_ESM.jpg]

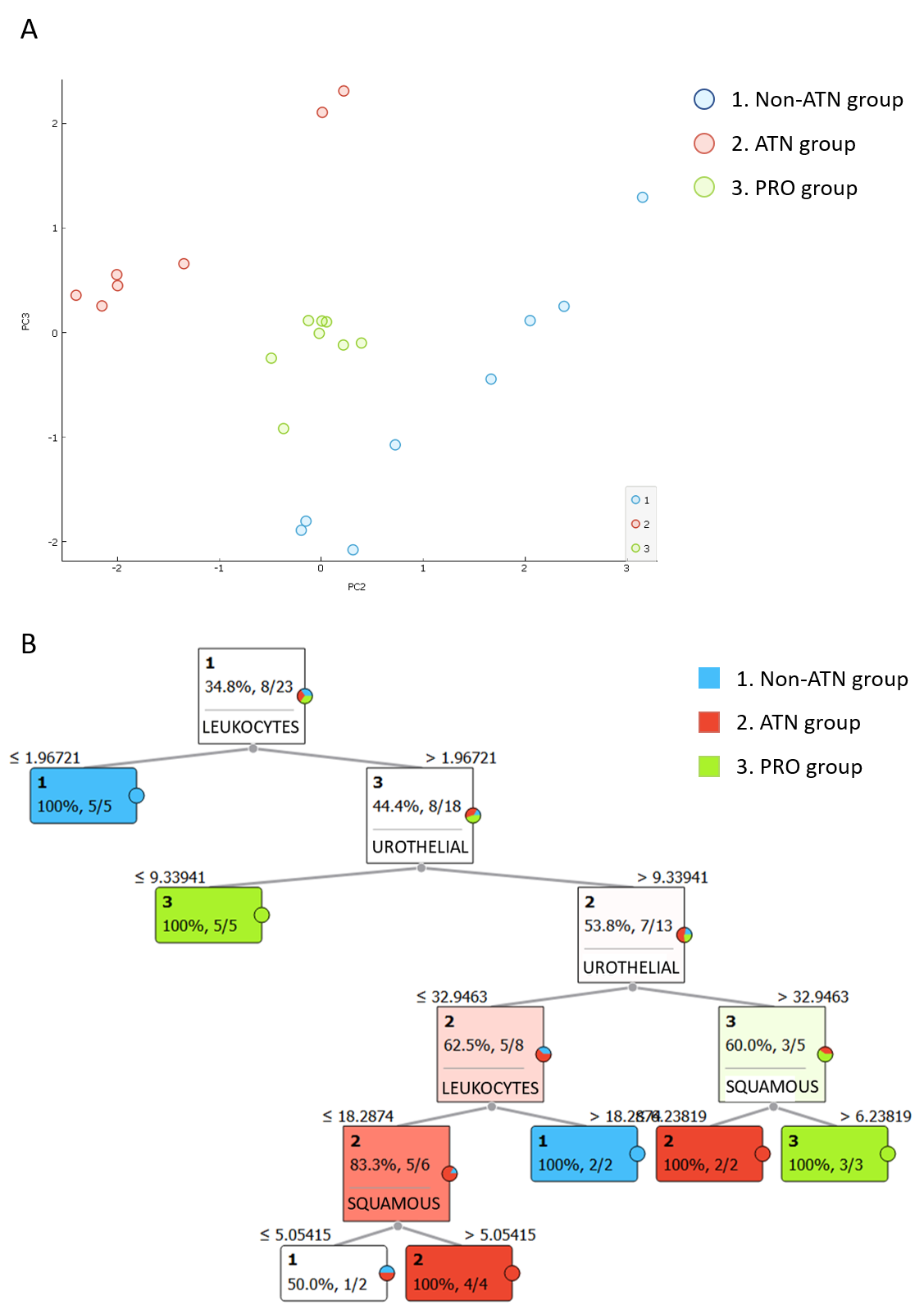

Supplement: Supplementary file 3 — Additional file 3: Figure S2. Algorithmic models of patients without ATN or proliferative glomerular disease (Non-ATN – Group 1/blue), with ATN (ATN – Group 2/red) and with glomerular proliferative lesion (PRO – Group 3/green). (A) Principal component analysis of groups of patients based in AKI diagnosis and cell numbers and types. (B) Binary tree model of groups of patients based in cell numbers and types. Transition of colors means the classification of groups accordingly as follows: blue - Non-ATN/Group 1; red – ATN/Group 2 and green – PRO/Group 3. [file 13104_2020_5244_MOESM3_ESM.png]
