# Supplementary material for: Urinary cytology: a potential tool for differential diagnosis of acute kidney injury in patients with nephrotic syndrome
Source: BMC Res Notes. 2020 Aug 27;13:401. doi: 10.1186/s13104-020-05244-6 (PMC7453712; doi:10.1186/s13104-020-05244-6)
Supplement: Supplementary file 2 — Additional file 2: Table S1. Cross-validation of models in urine cytology for differential diagnosis of AKI. [file 13104_2020_5244_MOESM2_ESM.docx]

**Table S1.** Cross-validation of models in urine cytology for differential diagnosis of AKI.

| Model | AUC | Precision |
| --- | --- | --- |
| Tree | 0.864 | 0.909 |
| Random Forest | 0.815 | 0.837 |
| Logistic Regression | 0.864 | 0.778 |

Notes: Test performance applied to average over groups with or without AKI. AUC= area under ROC curve.
